# Growth characteristics of early-stage (IA) lung adenocarcinoma and its value in predicting lymph node metastasis

**DOI:** 10.1186/s40644-023-00631-1

**Published:** 2023-12-01

**Authors:** Mengxi Liu, Junhao Mu, Feipeng Song, Xiangling Liu, Weiwei Jing, Fajin Lv

**Affiliations:** 1 Department of Radiology, The First Affiliated Hospital of Chongqing Medical University, Chongqing, China; 2 Department of Respiratory and Critical Care Medicine, The First Affiliated Hospital of Chongqing Medical University, Chongqing, China

**Keywords:** Lung adenocarcinoma, Lymph node (LN) Metastasis, Solitary pulmonary nodule (SPN), Volume doubling time (VDT), Mass doubling time (MDT)

## Abstract

**Background:**

We aim to compare the differences in growth characteristics between part-solid and solid lung adenocarcinoma, and to investigate the value of volume doubling time (VDT) or mass doubling time (MDT) in predicting lymph node (LN) metastasis and preoperative evaluation in patients of early-stage (IA) non-small cell lung cancer (NSCLC).

**Method:**

We reviewed 8,653 cases of surgically resected stage IA lung adenocarcinoma between 2018 and 2022, with two follow-up visits at least 3 months apart, comparing diameter, volume, and mass growth of pSN and SN. VDT and MDT calculations for nodules with a volume change of at least 25%. Univariable or multivariable analysis was used to identify the risk factors. The area under the curve (AUC) for the receiver operating characteristic (ROC) curves was used to evaluate the diagnostic value.

**Results:**

A total of 144 patients were included 114 with solid nodules (SN) and 25 with part-solid nodules (pSN). During the follow-up period, the mean VDTt and MDTt of SN were shorter than those of pSN, 337 vs. 541 days (*p* = 0.005), 298 vs. 458 days (*p* = 0.018), respectively. Without considering the ground-glass component, the mean VDTc and MDTc of SN were shorter than the solid component of pSN, 337 vs. 498 days (*p* = 0.004) and 298 vs. 453 days (*p* = 0.003), respectively. 27 nodules were clinically and pathologically diagnosed as N1/N2. Logistic regression identified initial diameter (*p* < 0.001), consolidation increase (*p* = 0.019), volume increase (*p* = 0.020), mass increase (*p* = 0.021), VDTt (*p* = 0.002), and MDTt (*p* = 0.004) were independent factors for LN metastasis. The ROC curves showed that the AUC for VDTt was 0.860 (95% CI, 0.778–0.943; *p* < 0.001) and for MDTt was 0.848 (95% CI, 0.759–0.936; *p* < 0.001).

**Conclusions:**

Our study showed significant differences in the growth characteristics of pSN and SN, and the application of VDT and MDT could be a valid predictor LN metastasis in patients with early-stage NSCLC.

## Introduction

Lung cancer is the second most common cancer and the leading cause of cancer-related deaths, with an estimated 1.8 million deaths worldwide [1], while lung adenocarcinoma is the most common type of lung cancer accounting for more than 50% of non-small cell lung cancer (NSCLC) [2]. With the popularity of low-dose computed tomography (LDCT), the detection rate of early-stage pulmonary nodules is gradually increasing in lung cancer screening [3–6]. Solitary pulmonary nodule (SPN) can be divided into solid nodule (SN) and subsolid nodule, which subsolid nodules can be further classified as pure ground-glass nodule (pGGN) and part-solid nodule (pSN) depending on whether the lung parenchyma is completely covered by nodules on CT imaging [7].

In the clinical follow-up of SPN, two-dimensional measurement of the maximum pulmonary nodules’ diameter is commonly used to determine the size of SPN, while three-dimensional measurements are more accurate than two-dimensional measurements [8, 9]. In addition, three-dimensional measurements can show the nodule growth or doubling time, which are important indicators for observing SPN growth. Although previous studies have shown the predictive value of volume doubling time (VDT) in screening patients for pulmonary nodules, however, the growth characteristics of pSN and SN, as well as their application in lymph node (LN) metastasis of early-stage lung adenocarcinoma have rarely been reported in the literature and lack experimental basis in clinical practice. Thus, the application and value of VDT or mass doubling time (MDT) in the preoperative evaluation of mediastinal LN is needed.

LN metastasis is not only the most common and major metastatic route in NSCLC, but also an important factor affecting clinical staging and prognosis [10–12]. N stage in NSCLC is critical for making treatment strategy decisions and determining clinical outcomes, and accurate assessment of LN metastasis is important for guiding surgical approaches or prognosis, because the surgical resection is the primary treatment option for patients with early-stage lung cancer [13]. The incidence of LN metastasis in patients with NSCLC is unknown, and the need for LN dissection during surgery is not clear preoperatively. Therefore, this study aims to provide an imaging basis for clinical diagnosis and preoperative evaluation by observing the growth trend of pulmonary nodules according to the follow-up of early-stage NSCLC, and the application of VDT and MDT in early-stage lung adenocarcinoma LN metastasis.

## Methods

### Patient selection

**Fig. 1:**
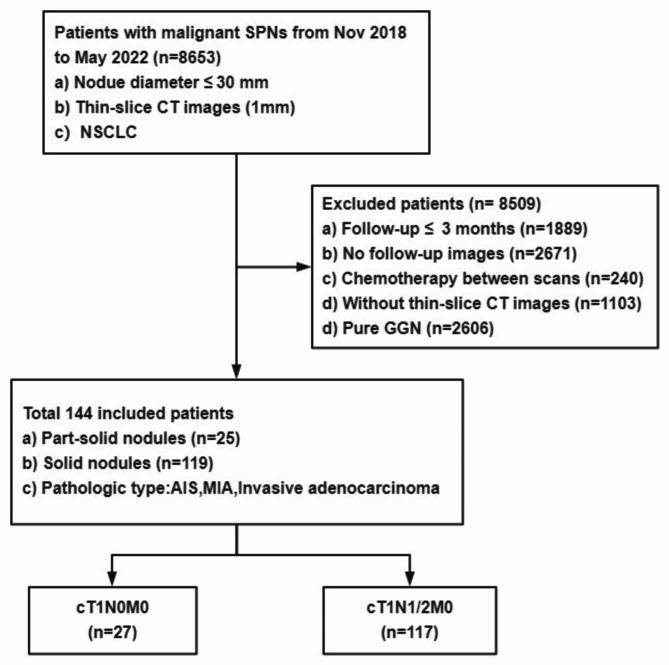
Patient selection. SPN: solitary pulmonary nodule; NSCLC: non-small cell lung cancer; GGN: ground-glass nodule; AIS: adenocarcinoma in situ; MIA: minimally invasive adenocarcinoma

This retrospectively study analyzed the clinical data of 8,653 patients who underwent thoracic surgical resection in the First Affiliated Hospital of Chongqing Medical University, China, from November 2018 to May 2022, and included pulmonary nodules with at least 3-month interval of preoperative follow-up. The interval of 1 or 2 days between the patient’s last CT examination and surgery. The inclusion criteria for SPN were as follows: (a) tumor diameter ≤ 3 cm; (b) systematic LN dissection that met the current standards (all LN stations 2–4 and 7–12 on the right side and stations 4–12 on the left side, according to the American Thoracic Society classification); (c) complete postoperative pathological data were available and the pathological findings showed adenocarcinoma. The exclusion criteria for malignant SPN were as follows: (a) benign pulmonary nodules; (b) history of preoperative adjuvant radiotherapy treatment, or patients with nodule volume reduction during follow-up; (c) follow-up ≤ 3 months or ≥ 12 months; (d) multiple pulmonary nodules. A total of 144 patients were enrolled in the study (Fig. 1).

### CT examinations

CT imaging of all subjects was performed on a 64-slice spiral CT scanner (SOMATOM Definition Flash, Siemens, Germany). The parameters of the non-contrast-enhanced CT examination were as follows: tube voltage, 110–120 kVp; tube current, 50–150 mAs; pitch, 1.0; detector collimation, 0.6 mm; rotation time, 0.5 s. CT images were reconstructed with a section thickness of 1.25 mm and viewed on standard lung window (width 1500 HU; level − 600 HU) and mediastinal window (width, 400 HU; level 30 HU) for image observation.

### Image analysis

All patients’ CT data were reviewed on a PACS workstation (Carestream Vue PACS) by two senior chest radiologists who were blinded to the pathological results. Interpretation discrepancy, if any, were resolved by consensus. The following radiological features are recorded during each patient’s initial examination and at follow-up process: (a) texture: SN was defined as the entire nodule consisting of solid components while pSN was defined as the ground-glass opacity (GGO) and the mixed density of solid components; (b) diameter: recorded as the mean of the longest diameter of the whole nodules; (c) solid component diameter: defined as a tumor lesion completely obscured the underlying bronchial structures and vascular markings from the lung window; (d) consolidation/tumor ratio (C/T): ratio of maximum solid diameter to maximum tumor diameter; (e) volume: measured automatically by picture archiving and communication system (Neusoft Medical Systems, Shenyang, China) after manual outlining of nodule margins slice by slice on thin-slice images; (f) density: obtained by freehand sketching the ROI along the tumor margin on workstation, averaging the 3 levels. CT attenuation in Hounsfifield Units (HU) can be translated directly into physical density in milligrams per milliliter by adding 1000 to the HU value [14]; (g) mass: defined as the product of volume and density of individual nodules; (h) LN metastasis: metastasis in systematic LN dissection, determined by postoperative pathology slides.

### VDT and MDT

First, we analyzed relative diameter, volume, and mass change for all the follow-up nodules, relative diameter change = (Dt − D0) / D0 × 100%; relative volume change = (Vt − V0) / V0 × 100%; relative mass change = (Mt- − M0) / M0 × 100%. D0, V0, and M0 represent the diameter, volume, and mass of the initial observation; Dt, Vt, and Mt are the diameter, volume, and mass of the follow-up time. We included nodules with ≥ 25% relative volume change in the VDT and MDT change statistics, as in previous studies [15, 16]. The VDT and MDT are calculated using the modified Schwartz formula [17]: VDT = [ln2 × ∆T] / [ln (Vt / V0)], where Vt and V0 represent the initial and follow-up volumes of the nodules, respectively, ∆T denotes the follow-up interval. Similarly, MDT = [ln2 × ∆T] / [ln (Mt / M0)], Mt and M0 represent the initial and follow-up masses of the nodules, respectively.

All nodules were staged according to the malignant tumor staging system in the eighth edition of the TNM Classification [18].

### Statistical analysis

All statistical analyses were performed using SPSS 22.0 statistical software (SPSS Inc., Chicago, Illinois, USA). Continuous variables were described by mean and standard deviation, while differences between two groups were compared by nonparametric tests or *t*-test. Categorical variables were expressed as percentages and differences between groups were compared by Chi-square test. Logistic regression equations were constructed to analyze the risk factors for LN metastasis in early-stage lung adenocarcinoma. Receiver-operating characteristic (ROC) curves was plotted to assess the diagnostic value of VDT and MDT in early-stage lung adenocarcinoma LN metastasis. Two-sided *p*-values less than 0.05 indicated statistical significance.

## Results

### Patient characteristics

There were 84 (58.3%) females and 60 (41.7%) males among the 144 patients, with the mean age of 59.18 ± 10.29 years (range, 32–78 years), and the mean tumor size of 11.95 ± 5.46 mm (range, 4.20-27.75 mm). We divided all patients into T1a (< 1 cm), cT1b (≥ 1 cm; < 2 cm), and cT1c (≥ 2 cm; ≤ 3 cm) according to the tumor size, the number of patients in each group was 52, 65, and 27, respectively. The percentage of SN in each group were 71.2%, 89.2%, and 88.9%, while the LN metastasis rates were 3.8%, 21.5%, and 40.7%, according to postoperative pathology, respectively. Table 1 summarizes the clinical data and pathological characteristics of 144 patients in each group. No significant differences were found in gender, family history of lung cancer, history of malignancy, smoking index, surgery, and location among the three groups (*p* > 0.05).

**Table 1:** Patient characteristics

Variable	T1a (n = 52)	T1b (n = 65)	T1c (n = 27)	*p*-value
Gender				0.124
Female	35(67.3)	32(49.2)	17(63)	
Male	17(32.7)	33(50.8)	10(37)	
Age (years)				0.000
< 60	40(76.9)	26(40.0)	12(44.4)	
≥ 60	12(23.1)	39(60.0)	15(55.6)	
Family history of lung cancer				0.129
No	49(94.2)	54(83.1)	25(92.6)	
Yes	3(5.8)	11(16.9)	2(7.4)	
History of malignancy				0.254
No	46(88.5)	62(95.4)	23(85.2)	
Yes	6(11.5)	3(4.6)	4(14.8)	
Smoking index				0.710
Nonsmoker	36(69.2)	38(58.5)	18(66.7)	
< 400	9(17.3)	10(15.4)	0(0)	
≥ 400	7(13.5)	17(26.2)	9(33.3)	
Surgery				0.827
Lobectomy	33(63.5)	43(66.2)	19(70.4)	
Segmentectomy	19(36.5)	22(33.8)	8(29.6)	
Location				0.191
Right upper lobe	15(28.8)	16(24.6)	7(25.9)	
Right middle lobe	4(7.7)	3(4.6)	4(14.8)	
Right lower lobe	10(19.2)	14(21.5)	2(7.4)	
Left upper lobe	12(23.1)	23(35.4)	5(18.5)	
Left lower lobe	11(21.2)	9(13.8)	9(33.3)	
Nodule types				0.025
pSN	15(28.8)	7(10.8)	3(11.1)	
SN	37(71.2)	58(89.2)	24(88.9)	
Pathological types				0.000
AIS	3(5.8)	0(0)	0(0)	
MIA	15(28.8)	4(6.2)	0(0)	
IA	34(65.4)	61(93.8)	27(100)	
LN Metastasis				0.000
No	50(96.2)	51(78.5)	16(59.3)	
Yes	2(3.8)	14(21.5)	11(40.7)	

pSN: part-solid nodule; SN: solid nodule; AIS: adenocarcinoma in situ; MIA: minimally invasive adenocarcinoma; IA: invasive adenocarcinoma; LN: lymph node

### Comparison of the growth characteristics of pSN and SN

Table 2 summarizes the CT features and follow-up changes of SPN. We divided them into pSN and SN for comparison, according to the imaging staging of pulmonary nodules. Among 25 pSN, the mean size was 12.03 ± 6.35 mm with a mean follow-up time of 170.4 ± 91.1 days, while the mean size was 14.28 ± 5.91 mm with a mean follow-up time of 180.5 ± 92.7 days among 119 SN.

**Fig. 2:**
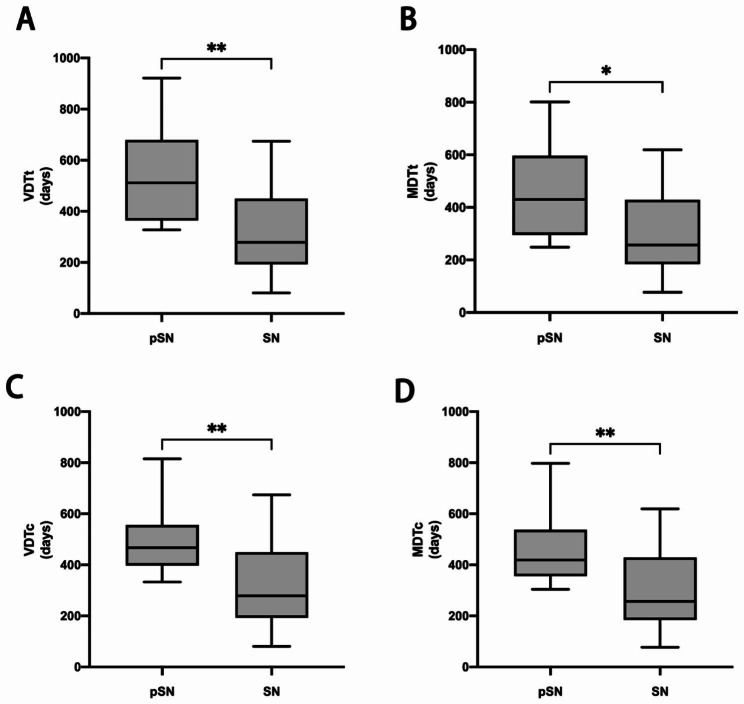
**(A-B)** Box plot shows VDT and MDT for the whole nodule of PSN and SN. The median VDTt and MDTt of SN was significantly shorter than pSN. (C-D) Box plot shows the solid component of PSN and SN. The median VDT and MDT of SN was significantly shorter than the solid component of pSN. ∗ represent *p* < 0.05. ∗∗ represent *p* < 0.01. pSN, part-solid nodules. SN, solid nodules. VDTt, volume doubling time of the whole nodule. MDTt, mass doubling time of the whole nodule. VDTc, volume doubling time of the solid component. MDTc, mass doubling time of the solid component

The changes in diameter, volume, and mass during the follow-up period of pSN and SN were statistically significant. The mean diameter, volume, and mass increases in the pSN and SN were 0.95 vs. 2.15 mm (*p* < 0.001), 195.31 vs. 696.25mm^3^ (*p* < 0.001), and 0.20 vs. 0.77 g (*p* < 0.001), respectively. The relative diameter, volume, and mass change during follow-up period were greater in the SN compared to pSN, 19.29% vs. 10.18% (*p* < 0.001), 45.85% vs. 17.12% (*p* < 0.001), and 51.78% vs. 22.71% (*p* < 0.001), respectively. 80 of all follow-up nodules exhibited growth, and we calculated volume doubling time of the whole nodule (VDTt) and mass doubling time of the whole nodule (MDTt) for these growing nodules. The VDTt for pSN and SN were 541 vs. 337 days (*P* = 0.005), while the MDTt was 458 vs. 298 days (*p* = 0.018), respectively (Fig. 2).

### Comparison of the growth characteristics of the solid component of pSN and SN

Without considering the ground-glass component, we compared the growth difference between the solid component of pSN and the SN, SN showed faster growth compared to the solid component of pSN (Table 2). During the follow-up period, the mean diameter, volume and mass increase of the solid component of pSN and SN were 0.92 vs. 2.15 mm (*p* < 0.001), 58.23 vs. 696.25 mm^3^ (*p* = 0.002), and 0.45 vs. 0.77 g (*p* = 0.002), respectively. The relative diameter, volume and mass change of the solid component of pSN and SN were 19.29% vs. 15.72% (*p* = 0.260), 24.74% vs. 45.85% (*p* = 0.007), and 29.82% vs. 51.78% (*p* = 0.008), respectively, the relative solid component volume and mass change were statistically differences. Volume doubling time of the solid component (VDTc) and mass doubling time of the solid component (MDTc) were calculated for nodules exhibited growth, the VDTc for the solid component of pSN and SN was 496 vs. 337 days (*P* = 0.004), while the MDTc was 453 vs. 298 days (*p* = 0.003), respectively (Fig. 2).

**Table 2:** Growth features of pSN and SN

Variable	pSN (n = 25)	SN (n = 119)	*p*-value
C-Stage			0.187
cT1a	15(60)	49(41.2)	
cT1b	7(28)	56(47.1)	
cT1c	3(12)	14(11.8)	
LN Metastasis			0.000
No	25(100)	92(77.3)	
Yes	0(0)	27(22.7)	
Follow-up (days)	170.4 ± 91.1	180.5 ± 92.7	0.585
Initial diameter (mm)	12.03 ± 6.35	14.28 ± 5.91	0.089
Solid component (mm)			0.000
< 5	10(40)	4(3.4)	
≥ 5	15(60)	115(96.6)	
Initial volume (mm^3^)	1561.96 ± 2625.61	1663.28 ± 2014.60	0.829
Initial mass (g)	1.31 ± 2.50	1.59 ± 1.94	0.536
Diameter increase (mm)	0.95 ± 0.68	2.15 ± 1.70	0.000
Volume increase (mm^3^)	195.31 ± 340.14	696.25 ± 990.43	0.000
Mass increase (g)	0.20 ± 0.38	0.77 ± 1.08	0.000
Relative diameter change (%)	10.18 ± 8.63	19.29 ± 14.72	0.000
Relative volume change (%)	17.12 ± 11.37	45.85 ± 36.87	0.000
Relative mass change (%)	22.71 ± 13.77	51.78 ± 39.65	0.000
Solid component increase (mm)	0.92 ± 0.81	2.15 ± 1.70	0.000
Solid component volume increase (mm^3^)	58.23 ± 70.5	696.25 ± 990.43	0.002
Solid component mass increase (g)	0.45 ± 0.19	0.77 ± 1.08	0.002
Relative solid component change (%)	15.72 ± 11.1	19.29 ± 14.72	0.260
Relative solid component volume change (%)	24.74 ± 22.46	45.85 ± 36.87	0.007
Relative solid component mass change (%)	29.82 ± 23.45	51.78 ± 39.65	0.008

pSN: part-solid nodule; SN: solid nodule; LN: lymph node

### Relationship between different pathological types of nodules and LN Metastasis

**Fig. 3:**
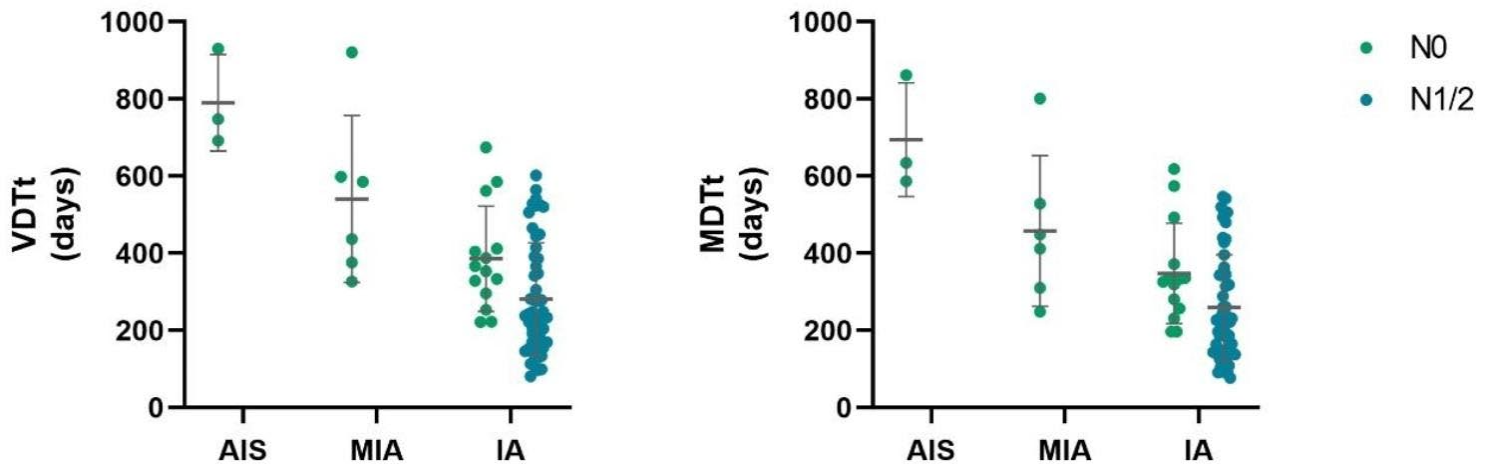
Graph shows the growth characteristics of the different pathological types of nodules in N0 and N1/2, where both AIS and MIA were N0. And both the mean VDTt and MDTt for IA were shorter in the N1/2 group than in N0, with the difference being statistically different (*p* < 0.05). AIS, adenocarcinoma in situ. MIA, minimally invasive adenocarcinoma. IA, invasive adenocarcinoma. VDTt, volume doubling time of the whole nodule. MDTt, mass doubling time of the whole nodule

Among the nodules we followed up, 3 of adenocarcinoma in situ (AIS) and 19 of minimally invasive adenocarcinoma (MIA) were all N0, while in invasive adenocarcinoma (IA), there were 95 N0 and 27 N1/2. The percentage composition of different pathological types in LN metastasis was statistically different (*p* = 0.039), Pearson chi-square test analysis showed a correlation between pathological type and LN metastasis with a column coefficient of 0.20, but according to the results of the regression analysis, pathology type was not an independent risk factor for LN metastasis (Table 3). We investigating the VDTt and MDTt of different pathological types of nodules, and found that both AIS and MIA were N0, with the VDTt of 790 days and the MDTt of 695 days for AIS, 541 and 458 days for MIA, respectively. The growth of IA showed significant differences in LN metastasis, with the VDTt of 386 vs. 248 days for the N0 and N1/2, respectively (*p* = 0.019), and the MDTt of 348 vs. 228 days for the N0 and N1/2, respectively (*p* = 0.034) (Fig. 3).

### Univariate and multivariate analysis for LN Metastasis

We divided all pulmonary nodules into two observation groups, N0 and N1/N2, according to the LN metastasis. Table 3 summarizes the patient characteristics and the results of the univariate analysis. Univariate analysis showed that gender (*p* = 0.04), smoking index (*p* = 0.037), nodule types (*p* = 0.008), pathological type (*p* = 0.039), initial diameter (*p* = 0.01), consolidation (*p* = 0.047), C/T ratio (*p* < 0.001), initial volume (*p* < 0.001), initial mass (*p* < 0.001), diameter increase (*p* < 0.001), consolidation increase (*p* < 0.001), volume increase (*p* < 0.001), mass increase (*p* < 0.001), relative diameter change (*p* < 0.001), relative volume change (*p* < 0.001), relative mass change (*p* < 0.001), VDTt (*p* = 0.001), and MDTt (*p* = 0.001) were all related to LN metastasis. On multivariate analysis, initial diameter (OR, 1.352; 95% CI, 1.141–1.584, *p* < 0.001), consolidation increase (OR, 0.543; 95% CI, 0.246–1.152, *p* = 0.019), volume increase (OR, 1.008; 95% CI, 1.001–1.030, *p* = 0.020), mass increase (OR, 1.019; 95% CI, 1.000-1.077, *p* = 0.021), VDTt (OR, 0.921; 95% CI, 0.878–0.965, *p* = 0.002), and MDTt (OR, 1.080; 95% CI, 1.017–1.134, *p* = 0.004) were independent factors for LNs metastasis (Table 4).

**Fig. 4:**
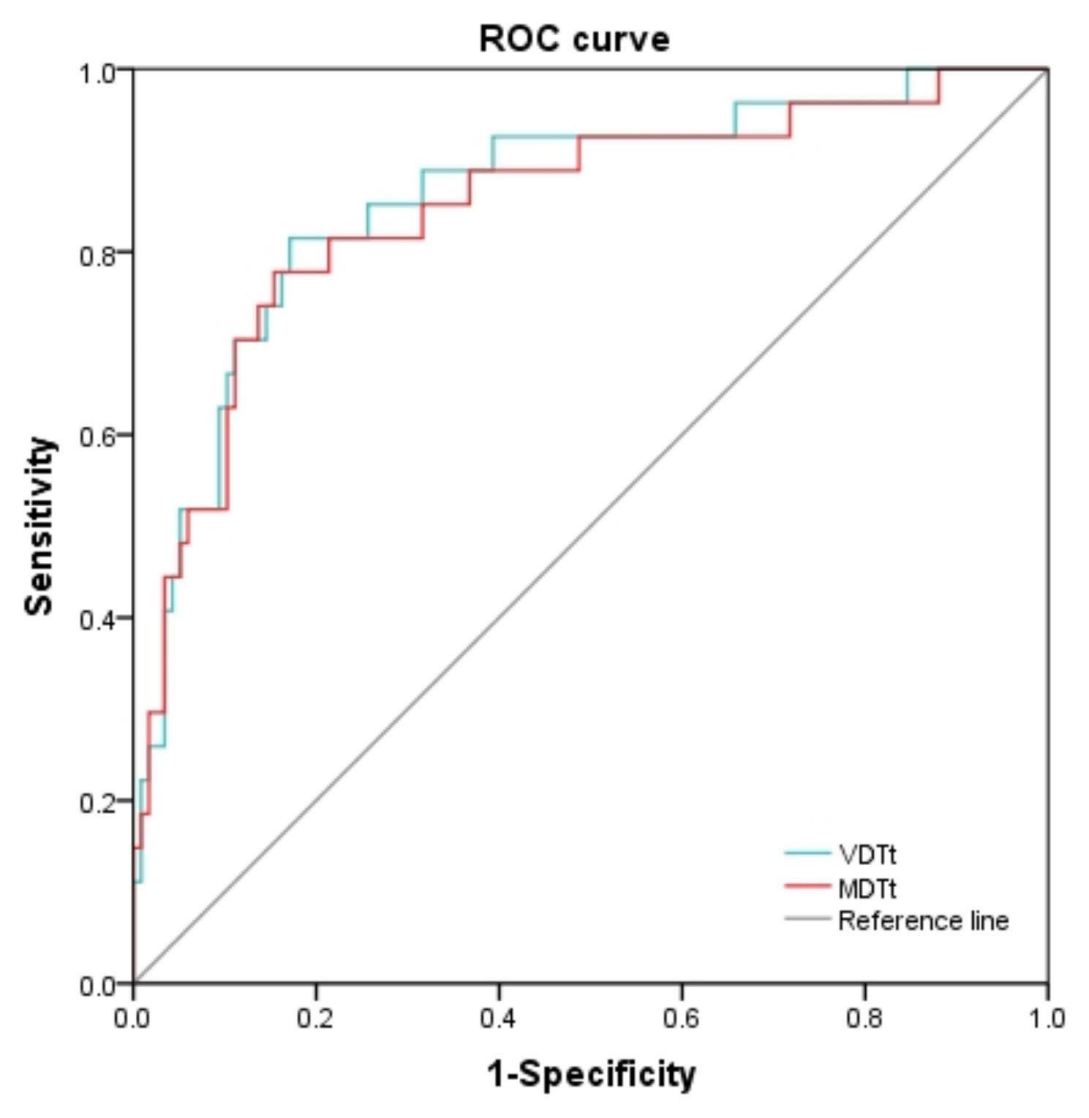
Receiver-operating characteristic curves. Reference line: (AUC 0.5); VDTt: volume doubling time of the whole nodule (AUC 0.860); MDTt: mass doubling time of the whole nodule (AUC 0.848). AUC: area under the curve

When drawing the ROC curve to evaluate the ability of VDTt and MDTt to predict the LN metastasis, the area under the curve (AUC) for VDTt was 0.860 (95% CI, 0.778–0.943; *p* < 0.001), if VDTt was used to predict the LN metastasis and assuming a threshold of 307 days for defining high risk for LN metastasis, with a sensitivity and specificity of 81.5% and 82.9%, respectively. The AUC for MDTt was 0.848 (95% CI, 0.759–0.936; *p* < 0.001), if MDTt was used to predict the LN metastasis and assuming a threshold of 254 days for defining high risk for LN metastasis, with a sensitivity and specificity of 77.8% and 84.6%, respectively (Fig. 4).

**Table 3:** Univariate analysis for metastasis

Variable	N0 (n = 117)	N1/N2 (n = 27)	*P*-value
Gender			0.040
Female	73(62.4)	11(40.7)	
Male	44(37.6)	16(59.3)	
Age (years)			0.261
< 60	66(56.4)	12(44.4)	
≥ 60	51(43.6)	15(55.6)	
Family history of Lung Cancer			0.675
No	107(91.5)	24(88.9)	
Yes	10(8.5)	3(11.1)	
History of malignancy			0.497
No	105(89.7)	23(85.2)	
Yes	12(10.3)	4(14.8)	
Smoking index			0.037
Nonsmoker	80(68.4)	12(44.4)	
< 400	20(17.1)	6(22.2)	
≥ 400	17(14.5)	9(33.3)	
Surgery			0.151
Lobectomy	74(63.2)	21(77.8)	
Segmentectomy	43(36.8)	6(22.2)	
Location			0.998
Right upper lobe	29(24.8)	7(25.9)	
Right middle lobe	9(7.7)	2(7.4)	
Right lower lobe	23(19.7)	5(18.5)	
Left upper lobe	32(27.4)	8(29.6)	
Left lower lobe	24(20.5)	5(18.5)	
Nodule type			0.008
pSN	25(21.4)	0(0)	
SN	92(78.6)	27(100)	
Pathological type			0.039
AIS	3(2.6)	0(0)	
MIA	19(16.2)	0(0)	
IA	95(81.2)	27(100)	
Follow-up (days)	176.4 ± 88.3	195.6 ± 107.3	0.395
Initial diameter (mm)	11.25 ± 5.21	14.95 ± 5.59	0.010
Solid component (mm)			0.047
< 5	14(12)	0(0)	
≥ 5	103(88)	27(100)	
C/T ratio	0.92 ± 0.19	1.0 ± 0.0	0.000
Initial volume (mm^3^)	1356.95 ± 1835.41	2896.88 ± 2785.87	0.010
Initial mass (g)	1.23 ± 1.69	2.87 ± 2.82	0.007
Diameter increase (mm)	1.43 ± 0.91	4.16 ± 2.18	0.000
Solid component increase (mm)	1.48 ± 1.10	4.10 ± 2.27	0.000
Volume increase (mm^3^)	302.96 ± 336.11	1936.66 ± 1415.01	0.000
Mass increase (g)	0.33 ± 0.37	2.09 ± 1.56	0.000
Relative diameter change (%)	14.63 ± 10.82	30.95 ± 19.32	0.000
Relative volume change (%)	29.85 ± 18.51	87.51 ± 51.36	0.000
Relative mass change (%)	34.76 ± 19.62	97.36 ± 54.70	0.000
VDTt (days)	424 ± 217	248 ± 167	0.001
MDTt (days)	378 ± 185	228 ± 158	0.001

pSN: part-solid nodule; SN: solid nodule; AIS: adenocarcinoma in situ; MIA: minimally invasive adenocarcinoma; IA: invasive adenocarcinoma; C/T ratio: the ratio of the maximum diameter of consolidation relative to the maximum tumor diameter; VDTt: volume doubling time of the whole nodule; MDTt: mass doubling time of the whole nodule

**Table 4:** Multivariate analysis for LN metastasis

Variable	OR	95%CI	*p*-value
Initial diameter	1.352	1.141–1.584	0.000
Consolidation increase	0.543	0.246–1.152	0.019
Volume increase	1.008	1.001–1.030	0.020
Mass increase	1.019	1.000-1.077	0.021
VDTt	0.921	0.878–0.965	0.002
MDTt	1.080	1.017–1.134	0.004

VDTt: volume doubling time of the whole nodule; MDTt: mass doubling time of the whole nodule; OR: odds ratio; CI: confidence interval

## Discussion

In our study, the overall prevalence of LN metastases was 18.7% (n = 27), and these N1/N2 were all SNs, accounting for 22.7% of the total SNs. There were no instances of LN metastases observed among the 25 pSNs. The solid component size, volume and mass increase were independent predictors of LN metastases. Additionally, the nodule VDTt and MDTt were considerably shorter in patients with LN metastases compared to those without metastases (248 vs. 428 days and 221 vs. 378 days, respectively).

**Fig. 5:**
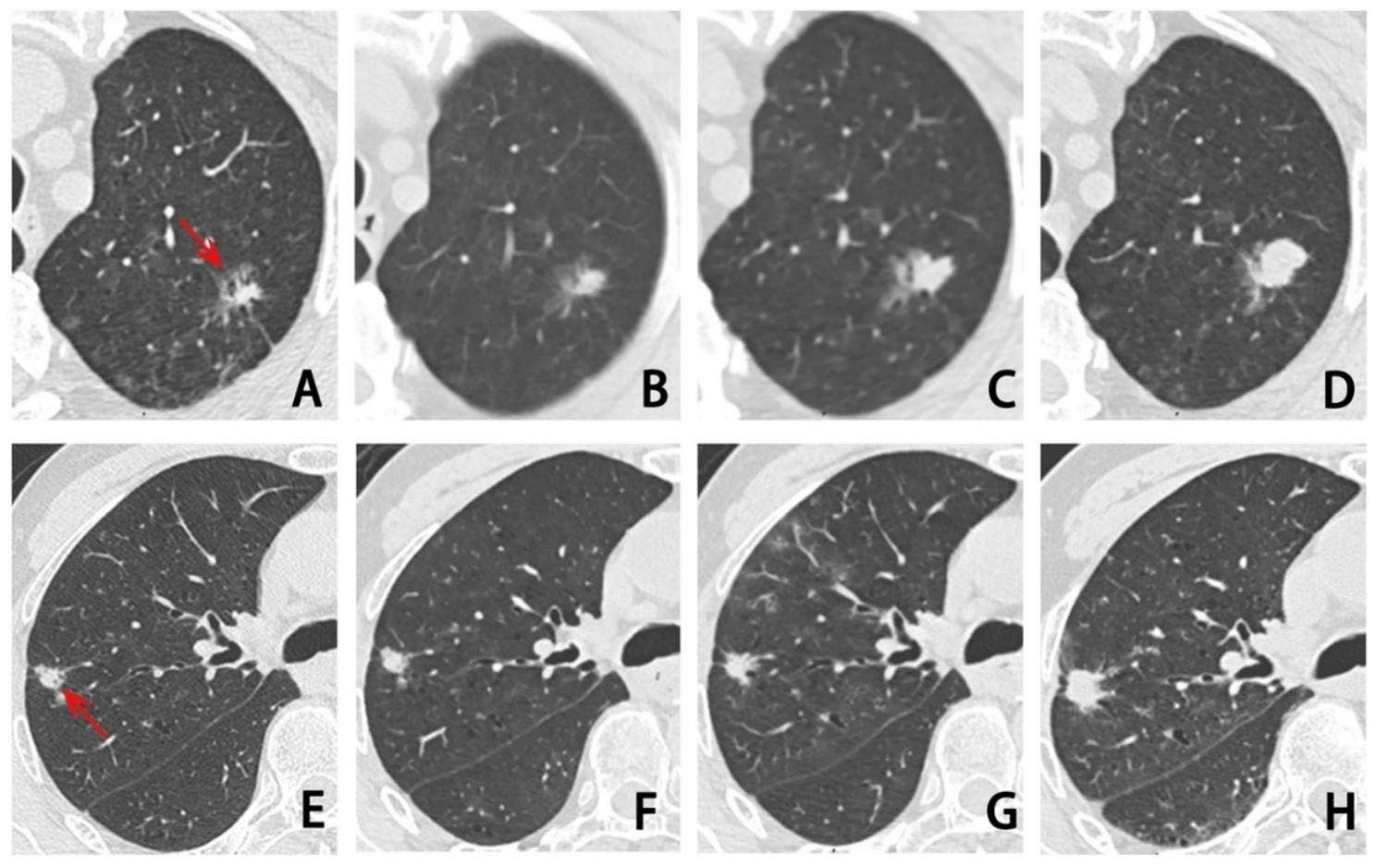
**(A-D)** pSN in the upper lobe of the left lung, IA, T1cN0M0, with a follow-up interval of 540 days, volume change rate 108.28%, VDTt 510 days, MDTt 427 days. **(E-H)** SN in the upper lobe of the right lung, IA, T1bN2M0, with a follow-up interval of 327 days, volume change rate 139.31%, VDTt 259 days, MDTt 249 days. The arrow indicates the nodule. pSN, part-solid nodule. SN, solid nodule. IA, invasive adenocarcinoma. VDTt, volume doubling time of the whole nodule. MDTt, mass doubling time of the whole nodule

The growth rates of pSN and SN exhibit disparities. Several studies on VDTt of pulmonary nodules have found that in malignant SPN, pSN usually has a VDTt of more than 400 days, whereas SN has a faster growth rate with the VDTt of 30–400 days [19–21]. Obayashi et al. [22] found that the median VDTt of adenocarcinoma with and without GGO was 725 and 177 days, respectively. The VDTt values for pSN and SN included in this study were 541 and 337 days, respectively (Fig. 5), which was consistent with the findings of their studies. SN has a shorter VDTt than pSN, most likely as a result of growth-related invasive components. Besides, Hoop et al. [23] found that a lower degree of variability when compared to the diameter or volume of malignant GGN resulted in an improved ability to use mass measures to identify nodule growth. This is consistent with our findings, which the mean MDTt lower than the mean VDTt for both pSN and SN.

Furthermore, pulmonary nodule growth can manifest itself not only as an increase in diameter or volume, but also as the appearance of a new solid component without significant volume change, or an increase in the internal solid component. Of the pSN in our investigation, 4 were MIA and 15 were IA, with 11 of them underwent solid component growth. While in all study cohorts, diameter growth (relative change ≥ 25%) occurred in 29 nodules, volume growth in 78 nodules, and mass growth in 96 nodules, with 2 nodules showing new solid components. The mean diameter, volume, and mass increase for the solid component of pSN and SN were 0.92 vs. 2.15 mm, 58.23 vs. 696.25 mm^3^, and 0.45 vs. 0.77 g, respectively. In addition, it was observed that SN exhibited shorter values for VDTc of 337 days and MDTc of 298 days in comparison to the solid component of pSN, which had VDTc and MDTc values of 496 days and 453 days, respectively.

This observation implies a potentially more aggressive biological behavior of SN, possibly due to their poorer pathological differentiation. It was observed that the distribution of pathological types in LN metastasis varied significantly, and a weak association (column coefficient = 0.20) was identified. All 27 LN metastasis nodules were SN with a pathological type of IA. However, none of the 15 patients with IA of the pSN exhibited LN metastasis. This finding can be attributed to the fact that the majority of pSN cases were largely composed of acinar adenocarcinoma (93.9%). In contrast, solid or micropapillary adenocarcinoma constituted a significant majority of 92.6% among the 27 SNs with LN metastases.

**Fig. 6:**
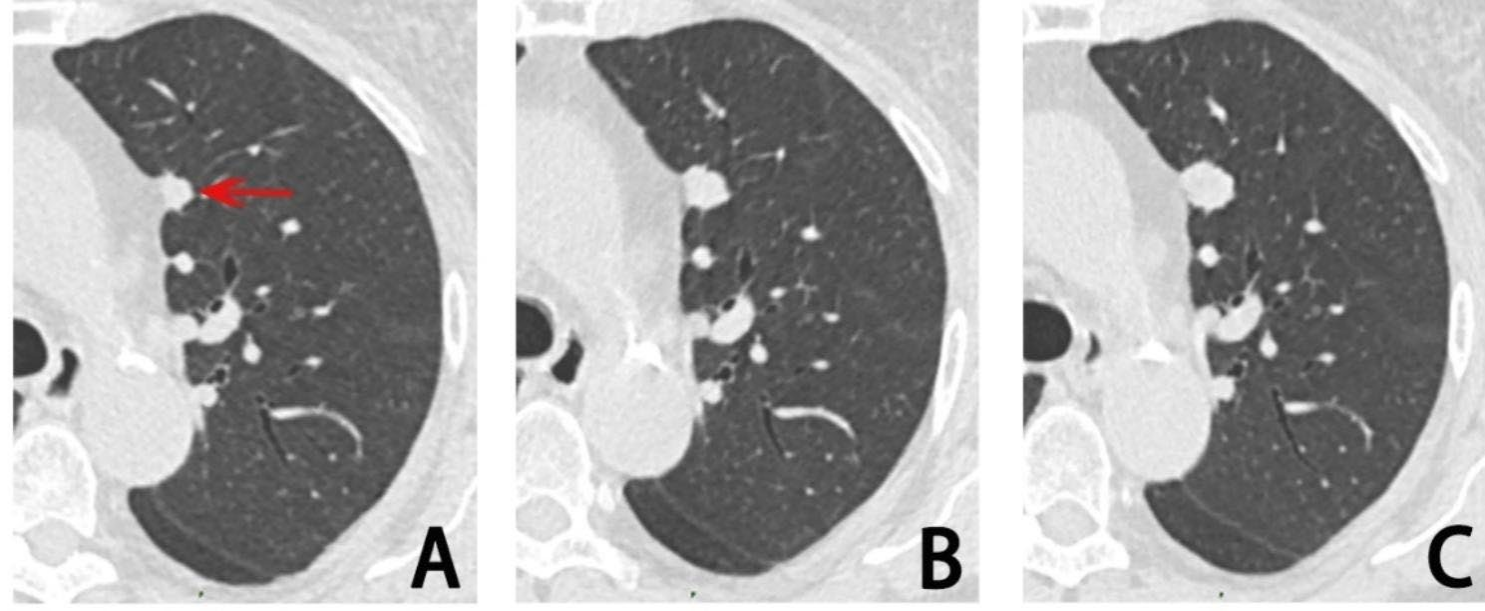
**(A-C)** SN in the upper lobe of the left lung, IA, T1bN1M0, follow-up interval 415 days, volume change rate 302.75%, VDTt 207 days, MDTt 199 days. The arrow indicates the nodule. SN, solid nodule. IA, invasive adenocarcinoma. VDTt, volume doubling time of the whole nodule. MDTt, mass doubling time of the whole nodule

The eighth edition of the TNM lung cancer classification incorporates the measurement of solid component size to determine the T stage of pSN, this is because the solid component corresponds to the invasive component [24]. Lee et al., [25] found that the size of the solid percentage was identified as a significant independent predictor based on the results of a multivariate analysis. The results in our study did not show the association between the solid component of pSN and LN metastasis, and this was due to the absence of pSN with LN metastases in our observed data. Regarding the SN, previous research has indicated a positive correlation between tumor diameter and the LN metastasis in individuals diagnosed with lung adenocarcinoma [25, 26]. In our study, the multivariate analysis of the SNs indicate that there is a significant association between tumor volume and mass growth and the probability of LN metastasis. Additionally, a shorter VDTt and MDTt are indicative of N1/2, implying that the presence LN metastasis is more probable in cases of rapidly growth SN (Fig. 6).

Because the status of LN determines the treatment strategy of the patient, it is particularly important to focus on accurate preoperative LN assessment in early-stage lung cancer. For the imaging evaluation of LNs metastasis, previous studies focused on the diagnostic efficacy of preoperative CT imaging in the LN metastasis [27, 28], and these studies have found correlations between mean CT values, maximum CT values, pleural depression, and lobar signs with the degree of tumor aggressiveness, as well as excellent differentiating accuracy (AUC, 0.823). In addition, carcinoembryonic antigen (CEA) levels, metabolic parameter LN-to-primary tumor ratio of SUVmax (LPR) are also important predictors of LN metastasis (AUC, 0.880) [29, 30]. While we innovatively explored the value of VDTt and MDTt in the preoperative assessment of LN, and according to the results of our study, the use of VDTt and MDTt to predict LN metastasis had better diagnostic efficacy (AUC, 0.860 and 0.848). Due to the heterogeneity of lung adenocarcinoma in terms of prevalence, racial differences, and tumor histology, there are limitations in comparing the parameters between studies. However, the inclusion of VDTt and MDTt in the follow-up of malignant SPN may improve the accuracy of predicting LN metastasis.

The present study has several limitations. First, we found a lack of follow-up information, including the type and number of nodules, in most LN metastasis nodules during the collection of imaging data, therefore some selection bias may apply. Secondly, we wanted to investigate the volume or mass changes in the follow-up of sub-centimeter nodules, which was not focused on in the present study. In addition, we did not compare the survival analysis of VDT in the high and low-risk groups for LN metastasis. A further prospective multicenter study is needed to assess not only the diagnostic ability but also the effect of VDT and MDT on making treatment decisions.

## Conclusions

For malignant SPN, there were differences in the growth characteristics of pSN and SN, with SN having a shorter VDTt and MDTt than pSN, and similarly, SN having a shorter VDTc and MDTc than the solid component of pSN, in which shorter intervals should be adopted for follow-up of SN to focus on nodule changes. In addition, the risk of LN metastasis was higher in SNs with shorter doubling times (VDTt < 307 days, MDTt < 254 days). Therefore, by assessing the growth characteristics of pulmonary nodules, VDT and MDT can be used to predict LN metastasis in early-stage NSCLC, and guide the selection of a clinical surgical approach and LN dissection.

## Data Availability

The datasets used and analyzed during the study are available from the corresponding authors on reasonable request.

## Abbreviations

AIS: Adenocarcinoma in situ
AUC: The area under the ROC curve
C/T ratio: Consolidation/tumor ratio
GGO: Ground-glass opacity
IA: Invasive adenocarcinoma
LDCT: Low-dose computed tomography
LN: Lymph node
MDTc: Mass doubling time of the solid component
MDTt: Mass doubling time of the whole nodule
MIA: Minimally invasive adenocarcinoma
NSCLC: Non-small cell lung cancer
pGGN: Pure ground-glass nodule
pSN: Part-solid nodule
ROC: Receiver operating characteristic
SN: Solid nodule
SPN: Solitary pulmonary nodule
VDTc: Volume doubling time of the solid component
VDTt: Volume doubling time of the whole nodule

## References

[1] Sung H, Ferlay J, Siegel RL, Laversanne M, Soerjomataram I, Jemal A, Bray F (2021). Global Cancer statistics 2020: GLOBOCAN estimates of incidence and Mortality Worldwide for 36 cancers in 185 countries. CA Cancer J Clin.

[2] Okayama H, Kohno T, Ishii Y, Shimada Y, Shiraishi K, Iwakawa R, Furuta K, Tsuta K, Shibata T, Yamamoto S, Watanabe S, Sakamoto H, Kumamoto K, Takenoshita S, Gotoh N, Mizuno H, Sarai A, Kawano S, Yamaguchi R, Miyano S, Yokota J (2012). Identification of genes upregulated in ALK-positive and EGFR/KRAS/ALK-negative lung adenocarcinomas. Cancer Res.

[3] Meza R, Meernik C, Jeon J, Cote ML (2015). Lung cancer incidence trends by gender, race and histology in the United States, 1973–2010. PLoS ONE.

[4] Aberle DR, Adams AM, Berg CD, Black WC, Clapp JD, Fagerstrom RM, Gareen IF, Gatsonis C, Marcus PM, Sicks JD (2011). Reduced Lung-cancer mortality with low-dose computed tomographic screening. N Engl J Med.

[5] de Koning HJ, van der Aalst CM, de Jong PA, Scholten ET, Nackaerts K, Heuvelmans MA, Lammers JJ, Weenink C, Yousaf-Khan U, Horeweg N, van ’t Westeinde S, Prokop M, Mali WP, Mohamed Hoesein FAA, van Ooijen PMA, Aerts J, den Bakker MA, Thunnissen E, Verschakelen J, Vliegenthart R, Walter JE, Ten Haaf K, Groen HJM (2020). Reduced Lung-Cancer mortality with volume CT screening in a Randomized Trial. N Engl J Med.

[6] Henschke CI, McCauley DI, Yankelevitz DF, Naidich DP, McGuinness G, Miettinen OS, Libby DM, Pasmantier MW, Koizumi J, Altorki NK, Smith JP (1999). Early Lung Cancer Action Project: overall design and findings from baseline screening. Lancet.

[7] Henschke CI, Yankelevitz DF, Mirtcheva R, McGuinness G, McCauley D, Miettinen OS (2002). CT screening for Lung cancer: frequency and significance of part-solid and nonsolid nodules. AJR Am J Roentgenol.

[8] Yankelevitz DF, Reeves AP, Kostis WJ, Zhao B, Henschke CI (2000). Small pulmonary nodules: volumetrically determined growth rates based on CT evaluation. Radiology.

[9] Revel MP, Lefort C, Bissery A, Bienvenu M, Aycard L, Chatellier G, Frija G (2004). Pulmonary nodules: preliminary experience with three-dimensional evaluation. Radiology.

[10] Rami-Porta R, Bolejack V, Crowley J, Ball D, Kim J, Lyons G, Rice T, Suzuki K, Thomas CF, Travis WD, Wu YL (2015). The IASLC Lung Cancer Staging Project: proposals for the revisions of the T descriptors in the Forthcoming Eighth Edition of the TNM classification for Lung Cancer. J Thorac Oncol.

[11] Asamura H, Chansky K, Crowley J, Goldstraw P, Rusch VW, Vansteenkiste JF, Watanabe H, Wu YL, Zielinski M, Ball D, Rami-Porta R (2015). The International Association for the study of Lung Cancer Lung Cancer Staging Project: proposals for the revision of the N descriptors in the Forthcoming 8th Edition of the TNM classification for Lung Cancer. J Thorac Oncol.

[12] Eberhardt WE, Mitchell A, Crowley J, Kondo H, Kim YT, Turrisi A 3rd, Goldstraw P, Rami-Porta R. The IASLC Lung Cancer Staging Project: proposals for the revision of the M descriptors in the Forthcoming Eighth Edition of the TNM classification of Lung Cancer. J Thorac Oncol. 2015;10(11):1515–22. 10.1097/jto.0000000000000673. 10.1097/JTO.0000000000000673 26536193

[13] Woodard GA, Jones KD, Jablons DM (2016). Lung Cancer Staging and Prognosis. Cancer Treat Res.

[14] Mull RT (1984). Mass estimates by computed tomography: physical density from CT numbers. AJR Am J Roentgenol.

[15] van Klaveren RJ, Oudkerk M, Prokop M, Scholten ET, Nackaerts K, Vernhout R, van Iersel CA, van den Bergh KA, van ’t Westeinde S, van der Aalst C, Thunnissen E, Xu DM, Wang Y, Zhao Y, Gietema HA, de Hoop BJ, Groen HJ, de Bock GH, van Ooijen P, Weenink C, Verschakelen J, Lammers JW, Timens W, Willebrand D, Vink A, Mali W, de Koning HJ (2009). Management of lung nodules detected by volume CT scanning. N Engl J Med.

[16] Zhang R, Tian P, Qiu Z, Liang Y, Li W (2020). The growth feature and its diagnostic value for benign and malignant pulmonary nodules met in routine clinical practice. J Thorac Dis.

[17] MacMahon H, Naidich DP, Goo JM, Lee KS, Leung ANC, Mayo JR, Mehta AC, Ohno Y, Powell CA, Prokop M, Rubin GD, Schaefer-Prokop CM, Travis WD, Van Schil PE, Bankier AA (2017). Guidelines for management of Incidental Pulmonary nodules detected on CT images: from the Fleischner Society 2017. Radiology.

[18] Goldstraw P, Chansky K, Crowley J, Rami-Porta R, Asamura H, Eberhardt WE, Nicholson AG, Groome P, Mitchell A (2016). The IASLC Lung Cancer Staging Project: proposals for revision of the TNM Stage groupings in the Forthcoming (Eighth) Edition of the TNM classification for Lung Cancer. J Thorac Oncol.

[19] Aoki T, Nakata H, Watanabe H, Nakamura K, Kasai T, Hashimoto H, Yasumoto K, Kido M (2000). Evolution of peripheral lung adenocarcinomas: CT findings correlated with histology and Tumor doubling time. AJR Am J Roentgenol.

[20] Kostis WJ, Yankelevitz DF, Reeves AP, Fluture SC, Henschke CI (2004). Small pulmonary nodules: reproducibility of three-dimensional volumetric measurement and estimation of time to follow-up CT. Radiology.

[21] Lindell RM, Hartman TE, Swensen SJ, Jett JR, Midthun DE, Mandrekar JN (2009). 5-year Lung cancer screening experience: growth curves of 18 Lung Cancers compared to histologic type, CT attenuation, stage, survival, and size. Chest.

[22] Obayashi K, Shimizu K, Nakazawa S, Nagashima T, Yajima T, Kosaka T, Atsumi J, Kawatani N, Yazawa T, Kaira K, Mogi A, Kuwano H (2018). The impact of histology and ground-glass opacity component on volume doubling time in primary Lung cancer. J Thorac Dis.

[23] de Hoop B, Gietema H, van de Vorst S, Murphy K, van Klaveren RJ, Prokop M (2010). Pulmonary ground-glass nodules: increase in mass as an early indicator of growth. Radiology.

[24] Aherne EA, Plodkowski AJ, Montecalvo J, Hayan S, Zheng J, Capanu M, Adusumilli PS, Travis WD, Ginsberg MS (2018). What CT characteristics of lepidic predominant pattern lung adenocarcinomas correlate with invasiveness on pathology?. Lung Cancer.

[25] Lee SY, Jeon JH, Jung W, Chae M, Hwang WJ, Hwang Y, Cho S, Chung JH, Kim K, Jheon S (2021). Predictive factors for Lymph Node Metastasis in Clinical Stage I Part-Solid Lung Adenocarcinoma. Ann Thorac Surg.

[26] Zhang Y, Sun Y, Shen L, Li Y, Xiang J, Zhang Y, Hu H, Chen H (2013). Predictive factors of lymph node status in small peripheral non-small cell Lung Cancers: Tumor histology is more reliable. Ann Surg Oncol.

[27] Ma J, Yang YL, Wang Y, Zhang XW, Gu XS, Wang ZC (2017). Relationship between computed tomography morphology and prognosis of patients with stage I non-small cell Lung cancer. Onco Targets Ther.

[28] Ichinose J, Kawaguchi Y, Nakao M, Matsuura Y, Okumura S, Ninomiya H, Oikado K, Nishio M, Mun M (2020). Utility of Maximum CT Value in Predicting the invasiveness of pure ground-glass nodules. Clin Lung Cancer.

[29] Haruki T, Aokage K, Miyoshi T, Hishida T, Ishii G, Yoshida J, Tsuboi M, Nakamura H, Nagai K (2015). Mediastinal nodal involvement in patients with clinical stage I non-small-cell Lung cancer: possibility of rational lymph node dissection. J Thorac Oncol.

[30] Nakanishi K, Nakamura S, Sugiyama T, Kadomatsu Y, Ueno H, Goto M, Ozeki N, Fukui T, Iwano S, Chen-Yoshikawa TF (2021). Diagnostic utility of metabolic parameters on FDG PET/CT for lymph node Metastasis in patients with cN2 non-small cell Lung cancer. BMC Cancer.

